# Core Competences of School Nurses for the Development of Anti-Bullying Strategies: Protocol for a Scoping Review

**DOI:** 10.3390/nursrep14040255

**Published:** 2024-11-13

**Authors:** Waldemar Brandão Neto, Helena Vitória Silva Pinheiro, Nicolle Augusta Artoni de Brito Araújo, Rayssa Cavalcanti Umbelino de Albergaria, Beatriz Molina Carvalho, Maria Regina Pontes Luz Riccioppo, Eunice de Fátima Soares da Cunha, Ana Virgínia Rodrigues Veríssimo, Estela Maria Leite Meirelles Monteiro, Maria Cândida de Carvalho Furtado

**Affiliations:** 1 Nursing Faculty Nossa Senhora das Graças, University of Pernambuco, Recife 52171-011, Pernambuco, Brazil; helena.pinheiro@upe.br (H.V.S.P.); nicolle.artoni@upe.br (N.A.A.d.B.A.); rayssa.albergaria@upe.br (R.C.U.d.A.); eunice.fatima@upe.br (E.d.F.S.d.C.); virginia.verissimo@upe.br (A.V.R.V.); 2 Maternal-Infant and Public Health Nursing Department, Ribeirão Preto College of Nursing, University of São Paulo, Ribeirão Preto 14040-902, São Paulo, Brazil; beatriz.molina.carvalho@usp.br (B.M.C.); maria.riccioppo@usp.br (M.R.P.L.R.); mcandida@eerp.usp.br (M.C.d.C.F.); 3 Nursing Department, Federal University of Pernambuco, Recife 50070-460, Pernambuco, Brazil; estela.monteiro@ufpe.br

**Keywords:** bullying, school nursing, professional competence, nurse’s role, school health services, school mental health services, scoping review

## Abstract

Background/Objectives: School nurses are capable of fostering safe and healthy school environments that are favorable to quality learning and social interactions. To this end, it is essential that they acquire a set of skills needed to implement bullying intervention programs. This article describes the protocol for a scoping review to identify and map the core competences for school nurses to develop anti-bullying strategies. Methods: The review will be conducted according to the JBI methodology for scoping reviews. The review will include primary, secondary, and gray literature, including theses and reports, found through comprehensive research in several databases: Scopus, WoS, APA PsycINFO, Embase, ScienceDirect, MEDLINE/PubMed, CINAHL (EBSCOhost), ERIC, LILACS, BDENF, IBECS, Cochrane Library, CAPES Dissertations and Theses Portal, RCAAP, Theses Canada, ProQuest Dissertations and Theses, and Google Scholar, as well as reference tracking. No geographical restrictions will be applied. The studies must include investigations into actions and interventions conducted by or involving nurses for the prevention of bullying in the school context. Two reviewers will act independently in screening the studies and extracting data using an extraction tool developed by the research team. Results: The results will be presented in a tabular format, supported by a narrative synthesis. The details of the scoping review will be reported according to the Preferred Reporting Items for Systematic Reviews and Meta-Analyses extension for Scoping Reviews guidelines. Conclusions: We anticipate that our scoping review will to strengthen a field of nursing that is still little explored, showing the school nurses’ role in prevent bullying episodes.

## 1. Introduction

Bullying is one of the main types of violence experienced by school-age children and adolescents around the world [1]. A global report by the United Nations Educational, Scientific and Cultural Organization (UNESCO) estimates that 246 million children and adolescents suffer some form of school violence and bullying every year [2]. A 2009–2010 survey (the Health Behavior in School-aged Children study) found a prevalence of exposure to bullying of more than 10% in the school-aged population [3]. Characterized by offensive, intentional, and repetitive behavior in unequal power relations between peers [4], bullying has negative repercussions throughout life, both for the victims and the aggressors. Research shows that exposure to bullying is related to a higher risk of depression [5] suicidal ideation and attempts [6], propensity to use substances [7], and low academic performance [8]. Therefore, anti-bullying interventions are not only necessary; they are also effective in reducing bullying rates and improving students’ mental health problems [9].

The presence of nurses in the educational environment has the potential to contribute to the early identification and prevention of this serious school health problem [10]. Evidence shows that students, teachers/management, and family members perceive nurses as an important figure in leading educational programs at school [11,12]. They offer, for example, conditions for the provision of playful and recreational activities and guidance and advice for the development of specific interventions aimed at preventing bullying within the school environment [13,14]. The nurse can also act as a facilitator of training programs for teachers and assist in the supervision of groups of students [15].

The National Association of School Nurses (NASN) defines school nursing as a specialized nursing practice that articulates health care and education for the optimal biopsychosocial development and well-being of students in the school environment [16]. Ethical and evidence-based school nursing practice should reflect collaborative work for school success and health and enable the school community to develop its full potential. The NASN has developed a theoretical framework with five non-hierarchical principles to guide such practices: care coordination, community/public health, leadership, quality improvement, and standards of practice, which bring together key actions for the development of health promotion programs, one of which is the prevention of school violence [17].

However, the implementation of school nursing practice faces challenges. The first of these relates to the lack of specific regulations governing the role of school nurses or whether to make the presence of nurses in educational institutions compulsory [18]. In some contexts, school nurse roles depend on primary health care professionals, as is the case in Brazil and other Latin American countries [19]. In countries such as the United States, Norway, England, and Australia, the nurse is at the center of the government’s strategy for a better psychosocial school environment [20]. The second issue, which is the focus of this study, is the need for specific knowledge, skills, and abilities, understood as the competences of the school nurse [21], to encourage nurses to engage in school violence prevention.

The development of professional competences makes it possible to build the skills needed to carry out health work, which include knowing, mobilizing, integrating, and transferring knowledge, resources, and skills [22]. In the context of school nursing, developing the skills needed to cope with school violence is crucial in order to demarcate a space for nurses to work and contribute to strengthening intersectoral education and health policies. Furthermore, these competences need to have a common basis to ensure effective and flexible work in order to meet the needs of each school’s reality in terms of structure, educational processes, and available resources.

The results of published reviews already show the relevance of nurses’ participation in the school setting and that of nursing interventions applied to bullying prevention [23,24]. However, no study has explored nurses’ willingness and preparedness to plan and implement anti-bullying interventions at school. To implement anti-bullying actions, it is essential to have training aimed at developing theoretical and practical skills that qualify as professional health practice [25]. A preliminary search in PROSPERO, MEDLINE, Cochrane Database of Systematic Reviews, Joanna Briggs Institute (JBI) Evidence Synthesis, and Open Science Framework (OSF) revealed no scoping reviews or current or ongoing systematic reviews on the subject, indicating a gap in research. Therefore, this scoping review aims to identify and map the core competences for school nursing in the development of anti-bullying strategies.

## 2. Review Questions

The research questions have been formulated based on prior reading of the existing literature that lists the main anti-bullying nursing interventions [26] and also through collaborative discussions with the research team, in which the PCC strategy was used: P = Population (nurses working in schools), C = Concept (core competences in school nursing), and C = context (anti-bullying strategies in schools).

The research question is as follows: what are the core competences for the performance of school nursing in the context of developing anti-bullying strategies?

This review also aims to answer the following specific questions:

(1) How do school nurses collaborate in anti-bullying strategies and what types of strategies/actions do they use the most?
(2) What skills and competencies does the school nurse have in preventing bullying?
(3) What are the benefits of the skills and competences identified for the school nurse’s role in bullying prevention?

## 3. Materials and Methods

**Figure 1:**
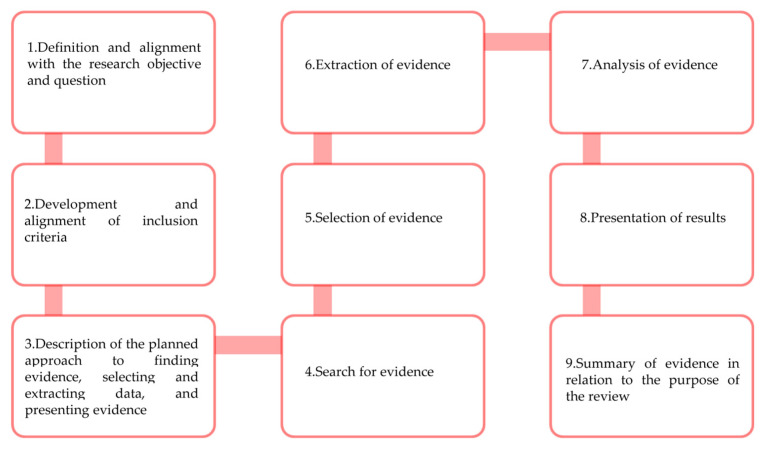
Stages to be followed in the scoping review according to Peters et al. (2020) [27].

This scoping review will follow the guidelines of the Joanna Briggs Institute (JBI) and will follow the steps proposed by Peters et al. (2020) [27] (Figure 1). The Preferred Reporting Items for Systematic reviews and Meta-Analysis extension for Scoping Reviews (PRISMA-ScR) guideline [28] will be used to structure the review report. The protocol for this review was registered on the Open Science Framework (OSF) platform (https://doi.org/10.17605/OSF.IO/DQ9JW) (accessed on 8 November 2024) to ensure the transparency and pioneering nature of this study.

### 3.1. Inclusion Criteria

To ensure the relevant selection of the literature and formulate the eligibility criteria, the PCC method (Population, Concept, Context) was used (Table 1).

### 3.2. Types of Sources

This scoping review will consider published and unpublished evidence. Primary studies, reviews, reports, commentaries, editorials, dissertations/theses, government guidelines and publications, and documents from scientific societies will be mapped. Books/book chapters and preprint documents will be excluded, as they have not undergone a peer review process, which may result in lower-quality published information. If conference papers and abstracts are considered eligible sources, the researchers will contact the respective authors to obtain a full report.

### 3.3. Search Strategy

The 3-stage search strategy will aim to locate published and unpublished sources. An initial limited search of MEDLINE/PubMed and CINAHL (EBSCOhost) with the keywords (bullying, school nursing, and prevention) was undertaken to identify articles on the topic. The analysis of the text words contained in the titles and abstracts of relevant articles, and the index terms/MeSH thesaurus used to describe these articles, was then used to develop a draft search strategy. A sample search strategy combining the controlled descriptors and keywords in all the databases is described in the Supplementary Material (Table S1). This strategy will be adapted for each database, such as the use of Emtree, MeSH, and DeCS controlled vocabulary when necessary. The search terms will further include derivatives of bullying (for example, *acoso escolar*, intimidation, school harassment, and school violence), school nursing (for example, public health nursing and family health nurses), and competence (for example, ability, manage, capacity, scope, and role). As recommended by the JBI methodology [31], the PCC structure will be used to categorize the relevant keywords related to the topic. It is worth mentioning that two reviewers and a librarian with experience in academic reviews in the health area collaborated in discussing the final search strategy, as well as assisting in the selection of databases. In the end, the authors will review the terms, Boolean operators, and results of the initial search to improve the search strategy.

The complete search will be carried out in the following databases: Scopus, Web of Science (WoS), APA PsycINFO, Embase, ScienceDirect, MEDLINE (EBSCO), CINAHL (EBSCOhost), ERIC, LILACS, BDENF, Índice Bibliográfico Español en Ciencias de la Salud (IBECS), and Cochrane Database of Systematic Reviews (Cochrane Library). In addition, the reference and citation lists of the included studies will be analyzed manually to screen for additional sources. This step aims to identify the existence of potential studies not previously captured.

Unpublished sources/gray literature (theses, dissertations, monographs, and manuals or guidelines from public bodies) will be searched through the CAPES/Brazil Theses and Dissertations Catalog, Scientific Open Access Scientific Repositories of Portugal (RCAAP), ProQuest Dissertations and Theses Global (PQDT), and Google Scholar (with screening of the first 20 pages of results), in addition to different scientific nursing societies and associations: the Virtual Library of the Conselho Federal de Enfermagem do Brasil (COFEN), the National Association of School Nurses (NASN), and the Asociación Madrileña de Enfermería en Centros Educativos (AMECE). The choice of the diversity of these databases is justified by the profile of a scoping review, which aims to include as many studies as possible [32].

Sources in any language will be included. If articles in languages other than English, Portuguese, and Spanish are eligible for full-text screening, they will be translated, if possible, with the assistance of Google Translate. No limits will be placed on the date of publication and geographical location.

### 3.4. Study Selection

Once the searches have been carried out, the citations will be imported into EndNote v.2020.3 (Clarivate Analytics, Philadelphia, PA, USA), with duplicates removed. The records will then be transferred to Rayyan (Qatar Computing Research Institute, Doha, Qatar), which helps to organize, archive, and select the studies. A pilot test will be conducted on a random sample of 20 titles/abstracts. Two reviewers will screen these articles using the eligibility criteria. If necessary, the team will discuss disagreements and modify the eligibility criteria. The screening will begin when 70% or more agreement is reached.

Two reviewers will independently examine the other titles and abstracts of the articles for the inclusion criteria. Potentially relevant sources will be retrieved in full using institutional access via the Federated Academic Community (CAFe) or by email to the authors when necessary. The full text of potentially relevant evidence will be independently and blindly assessed in detail according to the inclusion criteria by two reviewers. If there is disagreement between the reviewers during the evaluation of the full text, a third reviewer will be called in to analyze and decide on the inclusion or exclusion of the respective article. The reasons for excluding sources of evidence that do not meet the inclusion criteria will be reported in the final scoping review. The results of the bibliographic search and the study inclusion process will be reported in full in the final review in a PRISMA flowchart [33].

### 3.5. Data Extraction

Data will be extracted from the articles included in the scoping review by two independent reviewers to reduce the chance of error and bias using a data extraction tool developed by the authors (Appendix A). The data will be extracted from the Rayyan QCR platform and will include specific details on the title of the study, country of origin, year of publication, type of research and methodological approach, types of nursing practices/activities, competences identified in the prevention of bullying, benefits of professional competences, and the main findings relevant to the review questions. During the process of extracting data from each source of evidence, the tool may be modified, if necessary, to detail the information. The modifications will be reported in the final scoping review. Any disagreements that arise between reviewers will be resolved through discussion or with additional reviewers.

The competencies identified in the literature will be related to the axes/domains of professional school nursing practice competencies already reported in the literature: 1—Clinical competence; 2—Cultural and ethical care competence; 3—Communication and interpersonal relationship competence; 4—Management and leadership competence; 5—Health advocacy competence; 6—Education and research competence [34,35,36,37]. This analysis will help structure a framework of core competencies. In addition, the competences that are identified to a lesser degree will receive important attention in the final review, as it will not only open the way for future research but also incorporate trends in care, management, teaching, and research into the role of the school nurse.

The methodological quality of the included studies will not be assessed. Unlike systematic reviews, scoping reviews do not include a mandatory stage for critical appraisal and risk of bias in the studies; their purpose is to comprehensively group a body of evidence as a way of pointing out gaps in knowledge, raising definitions and characteristics about a particular factor or concept, or even providing results that could be better explored by conducting a systematic review [32,38].

### 3.6. Data Analysis and Presentation

The data will be presented in visual form, tables and narrative synthesis. The data, presented in a tabular format, will include information on study authorship, country of origin, year of publication, methodological approach, and main results. In addition, descriptive statistics (percentages or total numbers) will be used to describe the characteristics of the publications. Additional data presentation styles can be incorporated, such as pie charts, bar charts, word clouds, and diagrams. The narrative description will include the definition of the competences needed by the school nurse to prevent bullying, as well as the theoretical frameworks used, the main school nursing activities identified and the context in which they take place (community or hospital level), and the implications for the future of school nursing interventions in bullying prevention.

The scoping review is due to be completed in December 2024, taking into account the stages of searching the databases, mapping, refining, and analyzing the data.

## 4. Limitations and Strengths

There are some potential limitations to this scoping review. The first of these is related to the restriction of gray literature sources from countries in Asia and Oceania. Therefore, country-based studies will not be excluded. To overcome these limitations, a comprehensive search of the peer-reviewed and gray literature will be adopted, rigorously following the JBI methodology and the Preferred Reporting Items for Systematic Review and Meta-Analyses extension for Scoping Reviews guideline. Additional sources will also be consulted, such as reports/guidelines drawn up by scientific nursing organizations in leading countries, the results of which are often not easily available to the public. This protocol includes eligibility criteria explicitly aligned with the Population, Concept and Context strategy and defined based on a prior reading of the literature on the topic, with the aim of better understanding the profile and current role of school nursing practices.

The second limitation refers to the non-existence of school nurses in some contexts, or even the lack of regulation of their work. It is known that many countries face a shortage of nurses, which compromises the quality of health services [39]. This will be an important issue that could affect the discussion of competencies at a global level. Therefore, the impact of these limitations will be considered, and new limitations that may arise will be addressed when reporting the final results. Moreover, it is hoped that this review will encourage future systematic reviews into the effectiveness of school nursing skills in preventing bullying. Ethical approval will not be required for the development of the scoping review.

## 5. Conclusions and Implications for Expected School Nursing Practices

Based on the synthesis of evidence, we hope to strengthen a field of nursing that is still little explored, showing the potential of school nursing to prevent bullying episodes. By identifying the competencies and skills of the school nurse and relating them to the domains of professional competencies reported in the literature, the proposed scoping review will provide useful insights as to the structuring/reformulation of the competency framework to guide the school nurse in bullying prevention actions. As far as is known, this will be the first review to look at the subject with the following aims: to signal to academic managers the requirements necessary for school nursing training to deal with the issue of school violence, and to support future studies aimed at better performance and preparation of nurses to act with confidence in combating bullying and promoting a safer and healthier school environment.

School nursing is an expanding field, and its regulation is not a reality in many countries. Even though there are few publications in this area, and there are variations in the context of the school nurse’s work, the review will make it possible to discuss the development of essential school nursing competencies for bullying prevention that are emerging on a global level.

The results will be disseminated at research group meetings and nursing education conferences and published in a peer-reviewed nursing journal. The completed scoping review will contribute to the conclusion of the first author’s post-doctoral internship.

## Figures and Tables

**Table 1:** Inclusion and exclusion criteria.

PCC Framework	Inclusion	Exclusion
P—Population	This review will consider all the literature that reports registered nurses with or without a specialty in school health and primary health care and/or community health nurses working in school health programs (the professional spectrum has been broadened since it is understood that the figure of the school nurse is not consolidated in many countries). Thus, a school nurse will be defined as a professional who carries out systematic care actions with students and teachers in the school environment, which is also in line with the National Association of School Nurses (NASN) [29].	This excludes nursing students, technical professionals who provide nursing care, or other health professionals.
C—Concept	The concept of school nursing competence will be anchored in the intersection between knowledge, skills, attitudes, and values concerning the real problems experienced at school, with the ability to offer the best response/solution, considering the available resources [30]. Therefore, this review will include studies that explore school nursing knowledge, skills, and practices, with a focus on preventing bullying among students, whether the nurse is leading intervention actions/programs or collaborating in certain activities.	Studies dealing with the knowledge, actions, and practices of school nurses in the prevention of harassment towards teachers will be excluded.
C—Context	Anti-bullying strategies implemented within the school context, in a participatory way or not (including schools in urban or rural areas), will be included. However, studies that discuss the importance of networked actions to identify and prevent bullying (primary, secondary, and tertiary levels) will be reviewed.	Studies that discuss strategies for preventing bullying in nurses’ work environments or outside the school context will be excluded. In addition, studies that discuss school nursing actions involving the context of technical, higher, and youth and adult education will also be excluded.

## Data Availability

The data presented in this study are available in the article and Supplementary Materials. For data supporting reported results, please contact the authors of this review.

## Supplementary Materials

The following supporting information can be downloaded at https://www.mdpi.com/article/10.3390/nursrep14040255/s1, Table S1: Supplementary file of preliminary pilot search strategy in all databases.

## Public Involvement Statement

The public was not involved in any aspect of this research.

## Guidelines and Standards Statement

This manuscript follows the Joanna Briggs Institute (JBI) [27] guidelines and the Preferred Reporting Items for Systematic Reviews and Meta-Analyses extension for Scoping Reviews (PRISMA-ScR) guideline [28].

## Appendix A. Draft Data Extraction Tool

**Evidence Source Details and Characteristics**	**Standardization**
Authorship	Detail study authorship
Database	According to origin during the selection process
Year of publication	Year the study was published
Country	Place of origin of the study
Objective	Detail study objective
Type of research and methodological approach	Detail the type and design of the research carried out
Study population and sample size	Detail the population and research participants
Level of nurse participation/intervention at school	Detail the level of participation and planning of nurses in anti-bullying interventions at school and the collaboration of other professionals. Mention the practical context of implementation (public or private school, urban or rural), as well as whether there were networked actions (primary, secondary, or tertiary level of prevention)
Main school nursing activities to prevent bullying	List which activities the school nurse has partially or fully developed in bullying prevention interventions
Core competences *	Specify the dimensions of competences identified in the work and interventions conducted by school nurses in the prevention of bullying
Benefits of professional competences	Detail the benefits of training in core competences to be incorporated into the practice of school nurses in the prevention of bullying
Implications for the future of school nursing interventions	Identify strengths and weaknesses for the implementation of anti-bullying interventions by school nurses
***** A theoretical matrix of competencies was previously drawn up to guide the categorization of the studies. This matrix was based on international documents on professional competencies in health promotion and guidelines from scientific nursing societies.	
