# Supplementary material for: Core Competences of School Nurses for the Development of Anti-Bullying Strategies: Protocol for a Scoping Review
Source: Nurs Rep. 2024 Nov 13;14(4):3505–14. doi: 10.3390/nursrep14040255 (PMC11587460; doi:10.3390/nursrep14040255)
Supplement: Supplementary file 1 [file nursrep-14-00255-s001.zip › Table S1. Search Strategy .pdf]

Table S1. Supplementary file of preliminary pilot search strategy in all databases.

Databases	Search strategy
MEDLINE (EBSCO)	P- POPULATION: #**1** ((School Nursing [MeSH Terms] OR “School Nurse” [All Fields] OR “School Health” [All Fields] OR “School Health Promotion” [All Fields] OR “School Health Services” [All Fields] OR “School Mental Health Services” [All Fields] OR “School-based Nursing Interventions” [All Fields])) #**2** ((“Primary Care Nursing [MeSH Terms] OR “Public Health Nursing” [All Fields] OR “Family Health Nurses” [All Fields] OR “Community Health Nursing “[MeSH Terms]))<br><br>#**3** #1 OR #2<br><br>C- CONCEPT: #**4** ((Professional Competence [MeSH Terms] OR “Ability” [All Fields] OR “Manage” [All Fields] OR “Capacity” [All Fields] OR “Role” [All Fields] OR “Expertise” [All Fields] OR “Scope” [All Fields] OR “Competence” [All Fields] OR “Competency” [All Fields]))<br><br>C: CONTEXT #**5** ((“Bullying" [MeSH Terms] OR “School Violence” [All Fields] OR “School Harassment” [All Fields] OR “Intimidation” [All Fields] OR “Bullying Prevention” [All Fields] OR “Anti-bullying Programs” [All Fields] OR “Interventions Programs” [All Fields] OR “School Health Management Program” [All Fields]))<br><br>#**6** #3 AND (#4 OR #5)
CINAHL (EBSCOhost)	#**1** ((MH School Nursing* OR (“School Nurse” OR “School Health” OR “School Health Promotion” OR “School Health Services” OR “School Mental Health Services” OR “School-based Nursing Interventions”))<br>#**2** ((MH Primary Care Nursing* OR (“Public Health Nursing” OR "Family Health Nurses” OR “Community Health Nursing”))<br>#**3** ((MH Professional Competence* OR (“Ability” OR “Manage” OR “Capacity” OR “Role” OR “Expertise” OR “Scope” OR “Competence” OR “Competency”))<br>#**4** ((MH Bullying* OR (“School Violence” OR “School Harassment” OR “Intimidation” OR “Bullying Prevention” OR “Anti-bullying Programs” OR “Interventions Programs” OR “School Health Management Program”))
Web of Science (WoS)	#**1** ALL=((School Nursing*) OR (School Nurse) OR (School Health) OR (School Health Promotion) OR (School Health Services) OR (School Mental Health Services) OR (School-based Nursing Interventions))

	**#2** ALL=((Primary Care Nursing) OR (Public Health Nursing) OR (Family Health Nurses) OR (Community Health Nursing))<br>**#3** ALL=((Professional Competence*) OR (Ability) OR (Manage) OR (Capacity) OR (Role) OR (Scope) OR (Expertise) OR (Competence) OR (Competency))<br>**#4** ALL=((Bullying*) OR (Bullying Prevention) OR (Anti-bullying Programs) OR (Interventions Programs) OR (School Health Management Program))
Scopus	TITLE-ABS-KEY (School Nursing* OR (School Nurse OR School Health OR School Health Promotion OR School Health Services OR School Mental Health Services OR School-Based Nursing Interventions) AND Primary Care Nursing OR (Public Health Nursing OR Family Health Nurses OR Community Health Nursing) AND Professional Competence* OR (Ability OR Manage OR Capacity OR Role OR Scope OR Expertise OR Competence OR Competency) AND Bullying* OR (School Violence OR School Harassment OR Intimidation OR Bullying Prevention OR Anti-bullying Programs OR Interventions Programs OR School Health Management Program))
Embase	**#1** ('school nursing'/exp OR school nurse OR school-based nursing interventions)<br>**#2** ('public health nursing'/exp OR primary care nursing OR community health nursing)<br>**#3** ('competence'/exp OR 'professional competence'/exp OR competency OR 'expertise'/exp)<br>**#4** ('bullying'/exp OR school harassment OR bullying prevention OR 'intimidation '/exp)<br><br>**#5** (#1 OR #2) AND #3 AND #4
Science Direct	**#1** (school nursing OR School Nurse OR public health nursing)<br>**#2** (Primary Care Nursing OR Community Health Nursing)<br>**#3** (Professional Competence OR Ability OR Manage OR Capacity OR Expertise OR Role)<br><br>**#4** (#1 OR #2) AND #3
ERIC	(("School health" OR "School mental health services" OR "School nurse" OR "Public health nursing" OR "School-based nursing interventions") AND ("Bullying" OR "School violence" OR "School harassment" "Interventions programs" OR "Bullying prevention") AND ("Professional competence" OR "Competence" OR "Competency" OR "Ability" OR "Manage"))
APA PsycINFO	**#1** ((School nursing OR School nurse OR Public health nursing OR Primary care nursing OR Community health hursing) AND (School mental health services OR School-based nursing interventions OR School health promotion))<br>**#2** (Bullying OR School violence OR School harassment OR Interventions programs OR Bullying prevention)

	#**3** (Professional competence OR Competence OR Competency OR Ability OR Manage OR Role)<br><br>#**4** #1 AND #2 AND #3
Cochrane Library	#**1** (School Nursing) OR (School Nurse) OR (Public Health Nursing) OR (Family Health Nurses)<br><br>#**2** (School Mental Health Services) OR (Bullying) OR (School Violence) OR (School-based Nursing Interventions) OR (Interventions Programs)<br><br>#**3** #1 AND #2<br><br>#**4** (Professional Competence) OR (Competence) OR (Competency) OR (Ability) OR (Manage) OR (Capacity) OR (Role) OR (Expertise) OR (Scope)<br><br>#**5** #3 AND #4
LILACS/BDENF/ IBECS	#**1** ((Serviços de Enfermagem Escolar [DeCS] OR (School Nursing [DeCS] OR Servicios de Enfermería Escolar [DeCS] OR Escolar Soins infirmiers en milieu scolaire [DeCS]) AND (Enfermeiros de Saúde Pública [DeCS] OR Nurses, Public Health [DeCS] OR Enfermeras de Salud Pública [DeCS] OR Infirmières en santé publique [DeCS] OR Enfermeiros de Saúde Comunitária [DeCS] OR Nurses, Community Health [DeCS] OR Enfermeros de Salud Comunitaria [DeCS] OR Infirmières en santé communautaire [DeCS] OR Enfermagem de Atenção Primária [DeCS] OR Primary Care Nursing [DeCS] OR Enfermería de Atención Primaria [DeCS] OR Soins infirmiers de première ligne [DeCS]) AND (Violência Escolar [DeCS] OR School Violence [DeCS] OR Violencia Escolar [DeCS] OR Violence à l'école [DeCS] OR Bullying [DeCS] OR Acoso Escolar [DeCS] OR Brimades [DeCS]))<br><br>#**2** ((Competência Profissional [DeCS] OR Professional Competence [DeCS] OR Competencia Profesional [DeCS] OR Compétence professionnelle [DeCS] OR OR Competência Cultural [DeCS] OR Cultural Competency [DeCS] OR Competencia Cultural [DeCS] OR Compétence culturelle [DeCS] OR Gestão em Saúde [DeCS] OR Health Management [DeCS] OR Gestión en Salud [DeCS] OR Gestion de la Santé [DeCS] OR Prática Profissional [DeCS] OR Professional Practice [DeCS] OR Práctica Profesional [DeCS] OR Pratique professionnelle [DeCS] OR Papel do Profissional de Enfermagem [DeCS] OR Nurse's Role [DeCS] OR Rol de la Enfermera [DeCS] OR Rôle de l'infirmier [DeCS]))<br><br>#**3** #1 AND #2

Scientific nursing societies websites	Search strategy
COFEN Library Brazil/NASN/ AMECE	#**1** (School Nursing OR School Nurse OR Public Health Nursing OR Primary Care Nursing OR Family Health Nurses)<br><br>#**2** (Primary Care Nursing OR Community Health Nursing OR School Health OR School Mental Health Services OR Bullying, School Violence)
**Grey literature**	**Search strategy**
The ProQuest Dissertation & Theses Global	("School Nursing" OR "School Nurse" OR "Public Health Nursing") AND ("Bullying" OR "School Violence") AND ("Professional Competence" OR "Ability" OR "Manage" OR "Role")
Google Scholar	("School Nursing" OR "School Nurse" OR "Primary Care Nursing") AND ("Bullying" OR "School Violence") AND ("Professional Competence" OR "Ability" OR "Manage" OR "Role")
CAPES Theses and Dissertations Catalog/RCAAP	(Enfermagem Escolar OR Enfermeiros de Saúde Escolar OR Enfermagem de Atenção Primária) AND (Bullying OR Violência Escolar) AND (Prática Profissional OR Competência Professisonal)
